# miR-145-5p inhibits the proliferation of glioma stem cells by targeting translationally controlled tumor protein

**DOI:** 10.7150/jca.65543

**Published:** 2022-02-28

**Authors:** Qiujian Zhang, Zhe Cheng, Lei Shi, Gengsheng Mao

**Affiliations:** 1 Department of Neurosurgery, The First Affiliated Hospital of Jinan University, Jinan University, Guangzhou 510630, Guang Dong, China.; 2 Department of Neurosurgery, The Second Affiliated Hospital of Bengbu Medical College, Bengbu Medical College, Bengbu 233017, Anhui, China.; 3 Department of Neurosurgery, Gusu School, Nanjing Medical University, The First People's Hospital of Kunshan, Suzhou 215300, China.; 4 Department of Neurosurgery, The Third Medical Center of PLA General Hospital, Beijing 100039, China.

**Keywords:** glioma stem cells, miRNAs, miR-145-5p, translationally controlled tumor protein, proliferation, apoptosis

## Abstract

Glioma stem cells (GSCs) have potential for proliferation, self-renewal, and differentiation-the properties that play decisive roles in the process of malignancy in glioma. MicroRNAs (miRNAs) have been shown to regulate the characteristics of cancer stem cells. In this study, we show that miR-145-5p, a recently discovered miRNA, is expressed at low levels in primary GSCs (pGSCs). Upregulation of miR-145-5p resulted in the inhibition of proliferation and increased apoptosis of pGSCs. Furthermore, its overexpression resulted in reduced expression of translationally controlled tumor protein (TCTP). Bioinformatics analysis and luciferase targeting assay revealed that miR-145-5p exerts its effects by directly targeting TCTP. The expression of TCTP was significantly upregulated in pGSCs, and its silencing suppressed the proliferation and increased the apoptosis of pGSCs. Moreover, upregulation of TCTP attenuated the effect of miR-145-5p overexpression on the viability and apoptosis of pGSCs. The results of *in vitro* studies were corroborated *in vivo* using an orthotopic mouse model. Taken together, these results suggest that miR-145-5p could be a novel therapeutic target for gliomas through the suppression of TCTP in GSCs.

## Introduction

Glioblastoma (GBM), WHO grade IV, is the most malignant tumor of the central nervous system 1. It is characterized by rapid proliferation, high invasion, and abundant angiogenesis, which are important factors affecting its progression and determining the prognosis of patients 2. For patients with GBM, a combination of treatments including surgery, radiotherapy, and chemotherapy, is not satisfactory, and the tumor has a recurrence rate of approximately 100%. Patients treated with surgery plus post-operative radiotherapy have a median survival time of 11 months, and those subjected to post-operative radiotherapy concurrently with or without the adjuvant temozolomide (TMZ) have a median survival time of 23 months 3. GBM has strong proliferative and invasive abilities; however, the main obstacle in its treatment is that it cannot be completely removed 4. Glioma stem cells (GSCs) are the main source of cells that initiate and maintain the growth of glioma, and play a decisive role in the recurrence of GBM 5.

MicroRNAs (miRNAs) are important endogenous small RNAs, about 21-23 nucleotide in length, which are involved in a variety of intracellular gene silencing processes, including inhibition of translation, mRNA degradation, and deadenylation. Almost half of the known miRNAs have been found to be localized in oncogene-related regions 6. In recent years, studies on the relationship between miRNAs and gliomas have attracted much attention; investigations on the relationship between miRNAs and GSCs being the root cause of glioma genesis, proliferation, differentiation, invasion, metastasis, drug resistance, and recurrence have become increasingly important 7. MiRNAs may act on downstream target genes or proteins through Notch, Wnt, Hedgehog, PI3K/Akt, and other signaling pathways to regulate the expression of oncogenes or tumor suppressor genes, thereby, affecting the cell cycle, apoptosis, proliferation, and differentiation of GSCs, and ultimately affecting the formation and self-renewal ability of gliomas 8,9.

In glioma, miR-34a was reported to act on the Notch signaling pathway to induce G/S phase arrest and increase cell apoptosis, inhibit the differentiation of GSCs, and reduce tumor formation 10. Jiang et al. confirmed that an miR-342-3p/ISL2 feedback loop could regulate the angiogenic ability of patient-derived GSCs 11. An miR-26a inhibitor enhanced the inhibitory effects of TMZ on intracranial growth of GSCs by decreasing the expression of AP-2α 12. Recently, we demonstrated that upregulation of miR-145 inhibited the invasion and migration of GSCs by downregulating the expression of ABCG2 13. Overexpression of miR-145 increased the chemosensitivity of GSCs to demethoxycurcumin, resulting in increased cell growth inhibition and apoptosis 14. Chen et al. showed that the promotion of the stemness of glioma by circPTN was through the sponging of miR-145-5p 15. However, the mechanism underlying the role of miR-145-5p in GSCs remains unclear.

This study was aimed at elucidating the mechanism behind the role of miR-145-5p in GSCs. We demonstrate that overexpression of miR-145-5p inhibits the proliferation of GSCs, increases apoptosis, and decreases the growth of GSC xenografts in a nude mouse model, possibly through targeting the expression of translationally controlled tumor protein (TCTP).

## Materials and methods

### Cells and materials

U87, SHG44, and 293T cells were purchased from National Collection of Authenticated Cell Cultures (Shanghai, China). agomiR-145-5p (micrON hsa-miR-145-5p agomir, cat.miR40000437-4-5), agomir NC (micrON agomir NCl, cat.miR4N0000001-4-5), and TCTP siRNA (si-h-TCTP_001, cat.siB11525134333-1-5) and its corresponding negative controls were designed and synthesized by RiboBio Co., Ltd. The TCTP overexpression vector was constructed as described briefly below. The DNA coding sequence of TCTP was amplified and subcloned between BamHI and EcoRI sites in the pCDNA3.1(+) vector (Invitrogen, Carlsbad, CA) to construct pcDNA3.1-TCTP.

### Primary glioblastoma stem cell culture

Human glioma tissues were collected from the patients pathologically diagnosed with GBM after obtaining their informed consent. Cells were subcultured in Dulbecco's modified Eagle's medium (DMEM) supplemented with 2% fetal calf serum (FCS) for 1 week, and then in DMEM supplemented with 10% FCS containing penicillin and streptomycin (NanJing KeyGen Biotech Co., Ltd., Nanjing, China). The glial origin of cells was confirmed by staining with anti-GFAP monoclonal antibody (NanJing KeyGen Biotech Co., Ltd.). CD133-positive cells were separated by positive magnetic cell separation (MACS) (Miltenyi Biotech, Bergisch-Gladbach, Germany). Thereafter, pGSCs were cultivated in a stem cell-permissive medium, HUXNF-90011 (Cyagen, Santa Clara, CA 95050, USA). This study was approved by the Clinical Research Ethics Committee of the First People's Hospital of Kunshan. Inclusion criteria: 1) GBM patients with confirmed pathology, 2) GBM patients were treated by multimodality therapy, 3) The informed consent of family members and patients was obtained. Exclusion criteria: 1) GBM patients with unconfirmed pathology, 2) GBM patients with incomplete data records, 3) The informed consent of family members and patients was not obtained. All the patients were provided full information regarding the risks and benefits of the treatments, and provided signed consent. Clinical data including age, sex, and Karnofsky Performance Scale (KPS) score are shown in **Table 1.**

### Cell transfection

Agomir could be transfected without any transfection reagent. Agomir (1 nmol) was added to 50 µL RNase Free H_2_O, used as a storage solution, at a concentration of 20 μmol/L. For 96-well plates, 8 × 10^3^ cells were suspended in 49 µL Opti-MEM (Thermo Fisher Scientific, Waltham, MA, USA) and 1 μL 20 μmol/L agomir was added to a final concentration of 400 nM; the mixture was gently shaken. After 4-6 h of transfection, 50 µL complete medium was added for further culture. Changes in the expression level of miRNA were detected 24-72 h after transfection. In another plate, a corresponding amount of agomir added was increased to make the final concentration reach 200 nmol/L. For transfection with TCTP siRNA and pcDNA3.1-TCTP, 2 × 10^5^ cells were seeded per well in a 12-well plate with 1 mL complete medium to ensure 70%-90% confluence on the day of transfection. Lipofectamine 3000 reagent (1.5 µL) and 1 µg of DNA were diluted in 50 µL Opti-MEM (Thermo Fisher Scientific, Waltham, MA, USA) and then 2 µL P3000™ reagent was added to the diluted DNA. The diluted DNA solution with the P3000 reagent was added to the diluted Lipofectamine 3000 reagent (1:1 ratio) and incubated at room temperature for 5 min. Thereafter, 100 µL of the resulting complex was added to cells in complete medium. The expression of TCTP was detected by western blot analysis after 48-72 h of transfection.

### Quantitative RT-PCR analysis

Total RNA was extracted from the treated cells using the TRIzol reagent (Sigma-Aldrich, St. Louis, MO, USA). The concentration of RNA was determined using the Nanodrop spectrophotometer (Nanodrop 2000, Thermo Fisher Scientific). Using miDETECT A Track^TM^ miRNA qPCR Kit (cat.C10711, RiboBio Co., Ltd.), a total of 1 μg RNA was mixed with miDETECT A Track^TM^ Uni-RT Primer, and the reaction was performed at 42 °C for 1 h and the mixture was incubated at 72 °C for 10 min. After the reaction, the obtained cDNA was placed on ice and stored at -20 °C. cDNA (2 μL) was mixed with miDETECT A Track^TM^ Uni-Reverse Primer (RiboBio Co., Ltd.) and miDETECT A Track hsa-miR-145-5p Forward Primer (cat.miRA0000437-1-100, RiboBio Co., Ltd.) or miDETECT A Track^TM^ U6 Forward Primer (cat.miRAN0002, RiboBio Co., Ltd.). The amplification conditions were as follows: 95 °C for 10 min, followed by 40 cycles of 95 °C for 2 s, 60 °C for 20 s, and 72 °C for 10 s. The comparative threshold cycle (Ct) value was calculated using the 2^-ΔΔCt^ method 6.

To evaluate mRNA expression, RNA was first reverse transcribed using Prime Script RT Master Mix Kit (cat.RR036Q, Takara, Tokyo, Japan). Then, qRT-PCR was performed using TB Green^®^ Premix Ex Taq™ (cat.RR420Q, Takara) on ABI PRISM 7500 Real-Time PCR instrument (Applied Biosystems, Foster City, CA). The following primers were synthesized by Sangon (Shanghai, China). TCTP: forward primer, 5′-GTT GCT CTC CTG GAC TAC CG-3′, reverse primer, 5′-TGG TAA GCA GCC AAT TA-3′; β-actin: forward primer, 5′-TGT CAC CAA CTG GGA CGA TA-3′, reverse primer, 5′-GGG GTG TTG AAG GTC TCA AA-3′. The amplification conditions were as follows: 95 °C for 30 s, followed by 40 cycles of 95 °C for 5 s and 60 °C for 34 s, and then 95 °C for 15 s.

### CCK-8 cell growth assay

Cells (3,000/well) were seeded in 96-well plates and incubated at 37 °C in an atmosphere of 5% CO_2_ for 1 day. The cells were then treated with agomir NC, miR-145-5p agomir, siRNA negative control (si-NC), TCTP siRNA (si-TCTP), pcDNA3.1-Control (p-Control), or pcDNA3.1-TCTP (p-TCTP) for the indicated time. Thereafter, 10 μL CCK-8 reagent was added per well and the plate was incubated at 37 °C for 4 h. Subsequently, the absorbance was measured at 450 nm using a microplate reader (Sunrise, Tecan, Austria).

### Western blot analysis

For western analysis, GSCs were homogenized in RIPA Lysis Buffer KGP702 (NanJing KeyGen Biotech Co., Ltd.). The proteins were separated using SDS-PAGE (GenScript, Nanjing, China) at 140 V for 50 min. After that, the proteins were transferred onto a PVDF membrane (GenScript) at 300 mA for 60 min and then incubated overnight with primary antibodies against TCTP (ab37506), Bcl-2 (ab32124), Bax (ab32503), cleaved Caspase-3 (ab32042), IDH1 (ab172964), MGMT (ab108630), or GAPDH (ab9485), followed by incubation with an anti-rabbit secondary antibody (ab205718) for 1 h at 37 °C. All the antibodies were from Abcam (Cambridge, UK). The reaction was detected with a hypersensitive ECL chemiluminescence Kit (Beyotime Biotechnology, Nanjing, China). The blot was scanned using a BIO-RAD Gel Doc XR+ (Bio Rad Laboratories, Inc., Hercules, CA, USA), and the density of bands was analyzed using the Quantity One imaging software 4.5 (Bio Rad Laboratories, Inc.).

### Luciferase targeting assay

TargetScan (http://www.targetscan.org/vert_50/) was used for bioinformatics analysis to predict the targets of miR-145-5p. A construct containing a fragment of the 3′-UTR of TCTP mRNA carrying the putative miR-145-5p binding sequence or a mutated 3′-UTR of TCTP was synthesized by GenePharma Co., Ltd. (Shanghai, China) and cloned into a firefly luciferase reporter construct pGL3 (Promega, Madison, USA). 293T cells were seeded in a 24-well plate and cotransfected with luciferase reporters and miR-145-5p agomir using Lipofectamine 3000 (Invitrogen, Thermo Fisher Scientific, Inc., Carlsbad, CA, USA). The transfected cells were harvested after 48 h and then analyzed using the Dual-Luciferase Reporter Assay System (Promega).

### *In vivo* orthotopic mouse model

Male nude mice (18-20 g/6-8-weeks-old) were housed and maintained according to guidelines set by affiliated Kunshan Hospital of Jiangsu University and randomly assigned to one of the following three groups: blank group without any treatment, agomir NC group, transplanted with pGSCs transfected with micrON agomir NC (control, cat.miR4N0000001-4-5, RiboBio Co., Ltd.), and miR-145-5p agomir group, transplanted with pGSCs transfected with micrON hsa-miR-145-5p agomir (cat.miR40000437-4-5, RiboBio Co., Ltd.). A 100 μL cell suspension of 5 × 10^6^ cells were subcutaneously injected into the flanks of nude mice in each group. The tumor volumes were determined using a Vernier caliper at different time points. The volume was calculated using the formula: V_(mm_^3^_)_ = (L×W^2^)/2, where V = volume, L = length, and W = width. The relative tumor volume (RTV) = *V_t_* / *V_0_* (*V_0_*, the measured tumor volume at day 0; *V_t_*, the measured tumor volume at each time point). The relative tumor proliferation rates T/C (%) were calculated using the following formula: T/C (%)=T_RTV_/C_RTV_*100%. The tumor growth inhibition rates (TGI %) were calculated using the following formula:TGI (%)=(1-treatment group tumor weight/control group tumor weight) *100%.

At the end of the study, all nude mice were euthanized and the xenografts were carefully dissected. The xenografts were weighed and further processed for western blot and IHC analyses. This study was approved by the Animal Ethical Protection Association of Affiliated First People's Hospital of Kunshan. In addition, the experiment was conducted in accordance with the NIH guidelines for the care and use of laboratory animals.

### Statistical analysis

Statistical analyses were performed using the SPSS 21.0 software (SPSS, Chicago, IL). Data are presented as means ± standard deviation (SD). The differences were determined using ANOVA with repeated-measures ANOVA. To compare the differences between two groups, independent sample *t*-test was performed. A value of P<0.05 was considered significant.

## Results

### Evaluation of miR-145-5p and TCTP expression in human primary GSCs (pGSCs)

**Figure 1:**
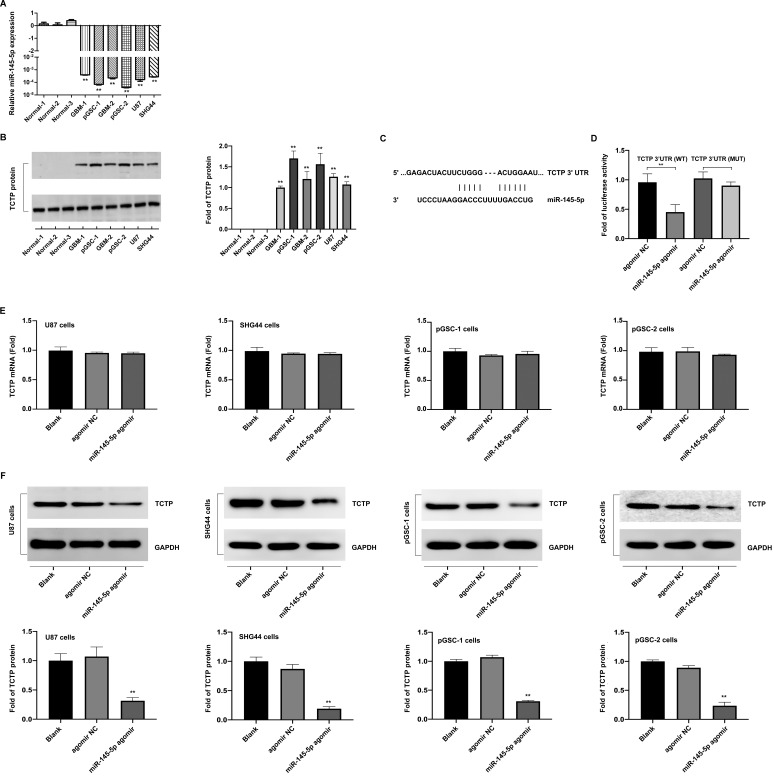
** The expression of miR-145-5p and translationally controlled tumor protein (TCTP) in human primary glioma stem cells (pGSCs).** Quantitative real-time PCR was performed to detect the expression of miR-145-5p (**A**) and TCTP (**B**) in human pGSCs. (**C**) Bioinformatics analysis of the seed sequence of miR-145-5p binding to the 3′-untranslated region (UTR) of TCTP mRNA. (**D**) A dual-luciferase reporter assay was used to confirm TCTP as a direct target of miR-145-5p. The change in TCTP expression was detected by quantitative real-time PCR (**E**) and western blot analysis (**F**). **p < 0.01.

The differences in the expression of miR-145-5p in normal brain, GBM-1, and GBM-2 tissues, and U87, SHG44, pGSC-1, and pGSC-2 cells were determined using qRT-PCR. The expression of miR-145-5p was low in GBM tissues and cell lines, and was the lowest in pGSCs (Fig. 1a). The expression of TCTP in the abovementioned tissues and cells was determined using western blot analysis and was found to be significantly overexpressed in GBM tissues and cell lines, being the highest in pGSCs (Fig. 1b). Considering the fact that the expression of TCTP correlated inversely with that of miR-145-5p in gliomas, we speculated that TCTP might be a target of miR-145-5p. Using the online bioinformatics tool, Targetscan, the potential binding sites for miR-145-5p in the 3′-untranslated region (UTR) of TCTP mRNA were explored (Fig. 1c). To further examine whether TCTP is a direct target of miR-145-5p, a dual-luciferase reporter assay was performed. As shown in Fig. 1d, a reduction in luciferase activity was observed in cells cotransfected with wild-type TCTP 3′-UTR (TCTP 3′UTR(WT)) and miR-145-5p agomir, but not in cells cotransfected with mutant-type TCTP 3′-UTR (TCTP 3′UTR(MUT)) and miR-145-5p agomir. These data confirmed that TCTP is a target of miR-145-5p. For further delineating the effects of miR-145-5p on the expression of TCTP, qPCR and western blot analysis were performed to detect the mRNA and protein levels of TCTP after transfection of cells with miR-145-5p. qPCR analysis showed that miR-145-5p did not influence the level of TCTP mRNA (Fig. 1e); however, it led to a significant decrease in the level of TCTP protein in U87, SHG44, pGSC-1, and pGSC-2 cells (Fig. 1f). These results suggested that miR-145-5p regulates the expression of TCTP at the post-transcriptional level.

### Upregulation of miR-145-5p results in the inhibition of proliferation of pGSCs and increased apoptosis *in vitro*

**Figure 2:**
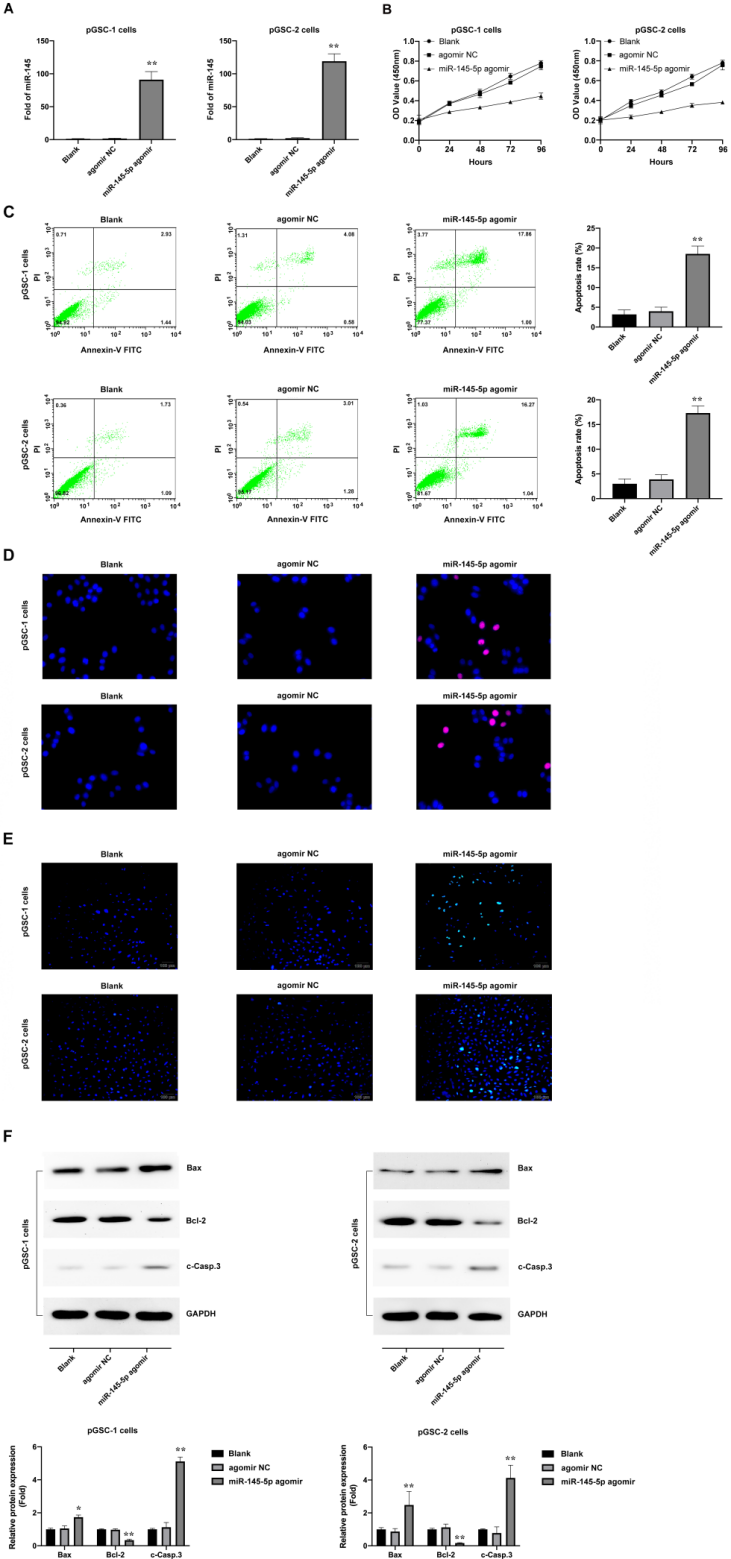
** miR-145-5p overexpression inhibits the proliferation of primary glioma stem cells (pGSCs) and increases the rate of apoptosis *in vitro*. (A)** Quantitative real-time PCR was performed to detect the expression of miR-145-5p in pGSCs transfected with miR-145-5p agomir. **(B)** CCK-8 assay was used to analyze the proliferation of pGSCs after transfection with miR-145-5p agomir. Apoptosis of pGSCs after transfection with miR-145-5p agomir was evaluated using Annexin V-FITC/PI double staining (**C**), TUNEL assay (**D**), and Hoechst 33342 staining (**E**). **(F)** Western blot analysis of the expression of Bcl-2, Bax, and c-Casp.3 in pGSCs after transfection with miR-145-5p agomir. *p < 0.05, **p < 0.01.

Loss of miR-145-5p was reported to promote cell proliferation and inhibition of apoptosis in laryngeal squamous cell carcinoma 16 and to inhibit migration and induce apoptosis in human non-small cell lung cancer cells 17. To evaluate the effects of miR-145-5p in GSCs, pGSCs were transfected with miR-145-5p agomir and the overexpression of miR-145-5p was verified by qRT-PCR (Fig. 2a). It was evident from the CCK-8 assay that the proliferation of pGSCs was suppressed after they were transfected with miR-145-5p agomir (Fig. 2b). Cell apoptosis was further evaluated using Annexin V-FITC/PI double staining (Fig. 2c), Hoechst 33342 staining (Fig. 2d), and TUNEL assays (Fig. 2e). Cells transfected with miR-145-5p agomir exhibited increased apoptosis rates compared with that of cells transfected with agomir negative control (NC) (Fig. 2c-e).

The expression of apoptotic proteins after transfection with miR-145-5p agomir was detected; a decrease in the expression of Bcl-2 and increase in the expression of Bax and cleaved Caspase-3 (c-Casp.3) was observed (Fig. 2f). Together, these results indicate that miR-145-5p induces the inhibition of cell proliferation and increases apoptosis of glioma cells *in vitro*.

### Upregulation of miR-145-5 inhibits the growth of pGSCs *in vivo*

**Figure 3:**
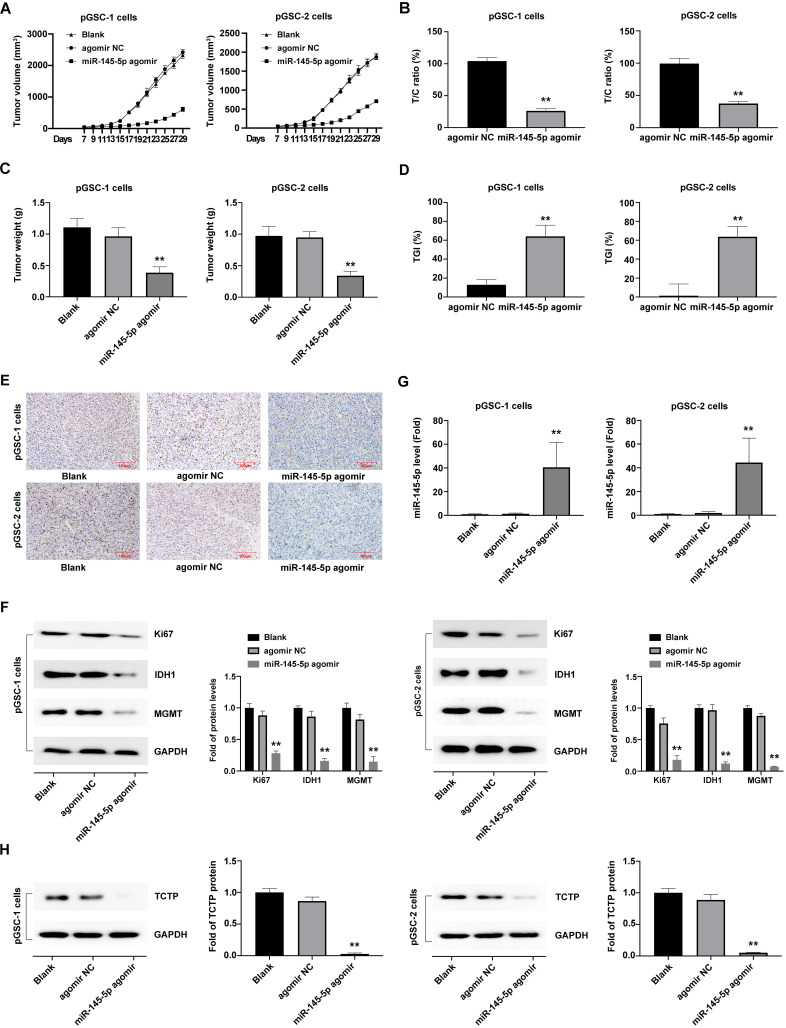
** Upregulation of miR-145-5 inhibits the growth of primary glioma cells (pGSCs) *in vivo*. (A)** miR-145-5p agomir decreased the size of tumors generated in a subcutaneously xenografted tumor model of pGSCs. **(B)** The relative tumor proliferation rate T/C (%) was calculated according to relative tumor volume (RTV). **(C)** Comparison of the weight of tumors generated by transplantation of pGSCs transfected with miR-145-5p agomir and of those transfected with agomir NC. **(D)** The tumor growth inhibition rates (TGI %) were calculated according to the data of the transplanted weight. **(E)** Decrease in the Ki67 ratio in the miR-145-5p agomir group observed by immunohistochemical (IHC) staining. **(F)** Expression of Ki67, IDH1, and MGMT detected by western blot analysis. **(G)** Expression of miR-145-5p in the xenograft tumor tissue analyzed by qRT-PCR. **(H)** Expression of translationally controlled tumor protein (TCTP) in xenograft tumor tissues assessed by western blot analysis. **p < 0.01.

Considering that miRNAs do not necessarily have the same effect *in vivo* and *in vitro*, the effects of miR-145-5p overexpression on tumorigenesis were further analyzed* in vivo*. The subcutaneously xenografted tumor model of pGSCs transfected with miR-145-5p agomir or agomir NC was established. The size of pGSC tumors in the miR-145-5p agomir transfection group was significantly smaller than that in the agomir NC group (Fig. 3a). According to relative tumor volume (RTV), the T/C ratio is commonly used to evaluate the anti-tumor activity. The lower T/C (%) represents the higher antitumor activity. As shown in Fig. 3b, the T/C (%) in miR-145-5p agomir group was 26.36% in pGSC-1 cells and 37.52% in pGSC-2 cells, which was significantly lower than that in agomir NC group (104.14% in pGSC-1 cells and 99.46% in pGSC-2 cells) (P<0.05). At the end of the experiment, the weight of the transplanted tumor is further evaluated. As shown in Fig. 3c, the weight of the tumor in miR-145-5p agomir group was less than that in agomir NC group. TGI is commonly used to evaluate the tumor growth inhibition rate. According to the data of the transplanted weight, we calculated TGI in miR-145-5p agomir and agomir NC. As shown in Fig. 3d, the TGI(%) in miR-145-5p agomir group was 64.12% in pGSC-1 cells and 63.68% in pGSC-2 cells, significantly lower than that in agomir NC group (12.95% in pGSC-1 cells and 1.37% in pGSC-2 cells) (P<0.05). These data suggested upregulation of miR-145-5 could efficiently inhibit the growth of pGSCs *in vivo*.

After that, the transplanted tumor specimens were collected for immunohistochemical (IHC) analysis. IHC analysis of the expression of Ki67, a proliferation marker, in tumor tissue revealed a decrease in the ratio of Ki67 expression in the miR-145-5p agomir groups compared with agomir NC and blank control group (P<0.05) (Fig. 3e). Furthermore, a significant decrease in the expression of Ki67, IDH1 and MGMT, was observed in the miR-145-5p agomir group (Fig. 3f).

Moreover, the expression of miR-145-5p in xenografted tumors developed after transplantation of cells transfected with miR-145-5p agomir, as detected by qRT-PCR analysis, was significantly high (Fig. 3g), which indicated that miR-145-5p was maintained at high levels for a long time in the transplanted tumor tissue. The TCTP levels, detected using western blot analysis, exhibited a decreasing trend in the miR-145-5p agomir group compared with those in the agomir NC group (P<0.05) (Fig. 3h). Taken together, these results clearly indicate that miR-145-5p also exerts anti-tumor effects in the transplanted tumors by targeting TCTP.

### Targeting of TCTP inhibits cell proliferation and induces cell apoptosis

**Figure 4:**
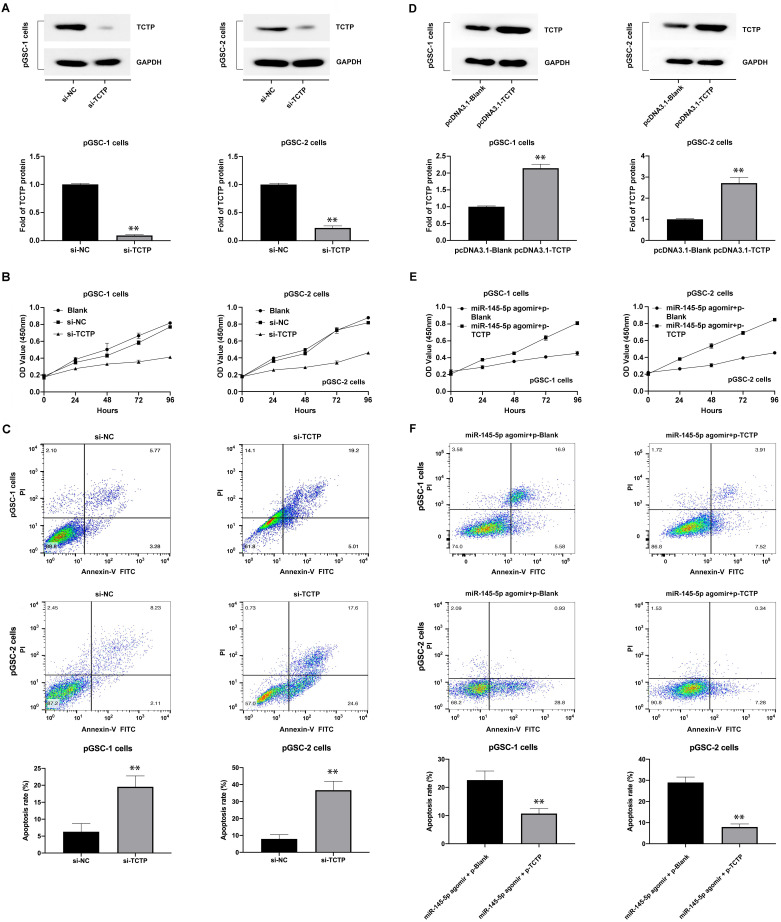
** Knockdown of translationally controlled tumor protein (TCTP) inhibits cell proliferation and induces apoptosis. (A)** Expression of TCTP in primary glioma stem cells (pGSCs) transfected with TCTP siRNA was assessed by western blot analysis. **(B)** Proliferation of pGSCs transfected with TCTP siRNA was examined by CCK-8 assay. **(C)** Apoptosis of pGSCs transfected with TCTP siRNA was examined by flow cytometry analysis. **(D)** Expression of TCTP in pGSCs transfected with pcDNA3.1-TCTP was detected by western blot analysis. **(E)** Proliferation of pGSCs transfected with miR-145-5p and/or pcDNA3.1-TCTP was detected by CCK-8 assay. **(F)** Apoptosis of pGSCs transfected with miR-145-5p and/or pcDNA3.1-TCTP was detected by flow cytometry analysis. **p < 0.01.

The effects of TCTP on the proliferation and apoptosis of GSCs were also analyzed. GSCs were transfected with TCTP siRNA (si-h-TPT1_001, cat. siB11525134333-1-5, RiboBio Co., Ltd., Guangzhou, China). Decreased expression of TCTP in GSCs was confirmed by western blot analysis (Fig. 4a). The proliferation and apoptosis of cells after transfection with the TCTP siRNA were examined using CCK-8 assay and Annexin V-FITC/PI double staining. The results showed that the downregulation of TCTP inhibited the proliferation of pGSCs (Fig. 4b) and caused an increase in apoptosis (Fig. 4c). Collectively, these data suggest that TCTP functions as a proto-oncogene in GSCs.

To determine whether the effects of miR-145-5p in anti-GSC activities were mediated by repressing TCTP, we first constructed a TCTP-overexpression vector lacking the 3′-UTR (pcDNA3.1-TCTP), according to the method described by Du et al. 18, and transfected it into GSCs. As shown in Fig. 4d, the expression of TCTP was obviously increased in pGSCs compared with that in the control group (P<0.05). Thereafter, the proliferation and apoptosis were detected after cotransfection of pGSCs with pcDNA3.1-TCTP and miR-145-5p agomir. As expected, cotransfection of pcDNA3.1-TCTP and miR-145-5p agomir significantly rescued the antiproliferative effects and apoptosis mediated by miR-145-5p agomir alone (Fig. 4e-f). These data clearly show that the antitumor effect of miR-145-5p on GSCs is in part mediated by targeting TCTP.

## Discussion

We found that miR-145-5p is an important miRNA, which regulates GSCs. The involvement of miRNAs, including miR-451, miR-18a, miR-363-3p, and miR-30b-5p, in regulating the formation and development of gliomas has been demonstrated in several studies. miR-451 was found to regulate the proliferation and invasion of glioma cells by targeting CAB39 19. miR-18a modulates the growth of glioma by targeting HMBOX1 20. miR-363-3p functions as a tumor suppressor in glioma by inhibiting pyruvate dehydrogenase B 21. miR-30b-5p inhibits the proliferation of glioma cells by targeting MTDH 22.

However, as of date, few studies have been conducted on the regulation of GSCs by miRNAs. Recently, increasing evidence has shown an abnormal expression of miRNAs in GSCs, and their key role in modulating the growth of GSCs. Thus, we believe that an understanding of the dysregulation of miRNA expression in GSCs would benefit the therapeutic targeting of gliomas. Rathod et al. showed low expression of miRNA-34a in GSCs, and reported that upregulation of the expression of this miRNA decreased the proliferative and migratory potential of GSCs by targeting the Akt and Wnt signaling pathways 23. In this study, we found that miR-145-5p is another important miRNA playing a central role in GSCs, with a lower expression in GSCs compared with that in normal brain and GBM tissues.

miR-145-5p was discovered recently. It is located on chromosome 9q31 and has five exons. It has the common attributes of miRNAs, being highly conserved and tissue-specific. Moreover, it participates in the regulation of proliferation, differentiation, and apoptosis of cells. The expression of miR-145-5p is significantly downregulated in a variety of tumor tissues, such as colon, lung, liver, and ovarian cancers, indicating that it is a tumor suppressor gene that plays an important role in the development of tumor, and may inhibit the occurrence of tumor through the same target 24-26. Du et al. reported that in gliomas, miR-145 induced apoptosis of glioma cells by targeting the BNIP3 and Notch signaling 27. Moreover, miR-145-5p was confirmed to downregulate the expression of Oct4, Sox2, and Klf4 in embryonic stem cells 28, and to inhibit the migration and invasion of GSCs by targeting ABCG2 29.

In the present study, we found that upregulation of miR-145-5p efficiently inhibited the proliferation of GSCs. Furthermore, luciferase targeting assay confirmed that miR-145-5p directly targets TCTP. TCTP is a multifunctional protein, which regulates cell growth, cell cycle, apoptosis, malignant transformation, and cancer progression 30. Glioma patients with higher expression of TCTP tend to have a shorter overall survival time 31. The overexpression of TCTP reduced the survival rate of glioma patients after treatment with radiotherapy and TMZ, and knockdown of TCTP could inhibit the proliferation of glioma cells *in vitro* and *in vivo* 32. We found that the inhibitory effect of miR-145-5p on the proliferation of GSCs could be attenuated by the overexpression of TCTP. These results indicate that miR-145-5p regulates the proliferation of GSCs by targeting TCTP.

## Conclusion

In conclusion, the role of miR-145-5p in GSCs was investigated in the present study. This miRNA was found to function as a tumor suppressor in GSCs. The mechanism underlying the regulation of the proliferation of GSCs by miR-145-5p involves the targeting of TCTP expression. These results suggest that miR-145-5p/TCTP might be a novel target for glioma treatment through the suppression of TCTP in GSCs.

## Figures and Tables

**Table 1:** Clinical characteristics of enrolled GBM patients

Clinical characteristics	GBM patient NO.1 (GBM-1)	GBM patient NO.2 (GBM-2)
Gender	Male	Female
Age	55	47
Tumor location	Frontal	Temporal
KPS	100	90
Tumor size (cm)	4.7*3.6*4.1	6.1*5.3*4.5
Modalities of treatment	Post-operative CCRT+ complete adjuvant TMZ	Post-operative CCRT+ complete adjuvant TMZ
WHO grade	WHO IV	WHO IV
